# Brace-Free Rehabilitation after Isolated Anterior Cruciate Ligament Reconstruction with Hamstring Tendon Autograft Is Not Inferior to Brace-Based Rehabilitation—A Randomised Controlled Trial

**DOI:** 10.3390/jcm12052074

**Published:** 2023-03-06

**Authors:** Christian Schoepp, Tobias Ohmann, Wolfgang Martin, Arthur Praetorius, Christine Seelmann, Marcel Dudda, Dirk Stengel, Jakob Hax

**Affiliations:** 1 Department of Arthroscopic Surgery, Sports Traumatology and Sports Medicine, BG Klinikum Duisburg gGmbH, Großenbaumer Allee 250, 47249 Duisburg, Germany; 2 Athletikum Rhein Ruhr, BG Klinikum Duisburg gGmbH, Großenbaumer Allee 250, 47249 Duisburg, Germany; 3 Department of Trauma, Hand and Reconstructive Surgery, University Hospital, Hufelandstraße 55, 45147 Essen, Germany; 4 Research Department, BG Klinikum Duisburg gGmbH, Großenbaumer Allee 250, 47249 Duisburg, Germany; 5 Department of Trauma and Reconstructive Surgery, BG Klinikum Duisburg gGmbH, Großenbaumer Allee 250, 47249 Duisburg, Germany; 6 BG Kliniken—Klinikverbund der Gesetzlichen Unfallversicherung gGmbH, Leipziger Pl. 1, 10117 Berlin, Germany; 7 Department of Knee and Hip Surgery, Schulthess Clinic, Lengghalde 2, 8008 Zurich, Switzerland

**Keywords:** knee, ACL, hamstring tendon, rehabilitation, bracing, brace-free

## Abstract

Purpose: The postoperative use of a rehabilitative knee brace after isolated primary anterior cruciate ligament (ACL) reconstruction (ACLR) using a hamstring tendon (HT) autograft is controversial. A knee brace may provide subjective safety but can cause damage if applied incorrectly. The aim of this study is to evaluate the effect of a knee brace on clinical outcomes following isolated ACLR using HT autograft. Methods: In this prospective randomised trial, 114 adults (32.4 ± 11.5 years, 35.1% women) underwent isolated ACLR using HT autograft after primary ACL rupture. Patients were randomly assigned to wear either a knee brace (*n* = 58) or no brace (*n* = 56) for 6 weeks postoperatively. An initial examination was performed preoperatively, and at 6 weeks and 4, 6, and 12 months. The primary endpoint was the subjective International Knee Documentation Committee (IKDC) score to measure participants’ subjective perceptions. Secondary endpoints included objective knee function assessed by IKDC, instrumented knee laxity measurements, isokinetic strength tests of the knee extensors and flexors, Lysholm Knee Score, Tegner Activity Score, Anterior Cruciate Ligament—Return to Sport after Injury Score, and quality of life determined by Short Form-36 (SF36). Results: There were no statistically significant or clinically meaningful differences in IKDC scores between the two study groups (3.29, 95% confidence interval (CI) −1.39 to 7.97, *p* = 0.03 for evidence of non-inferiority of brace-free compared with brace-based rehabilitation). The difference in Lysholm score was 3.20 (95% CI −2.47 to 8.87); the difference in SF36 physical component score 0.09 (95% CI −1.93 to 3.03). In addition, isokinetic testing did not reveal any clinically relevant differences between the groups (n.s.). Conclusions: Brace-free rehabilitation is non-inferior to a brace-based protocol regarding physical recovery 1 year after isolated ACLR using HT autograft. Consequently, the use of a knee brace might be avoided after such a procedure. Level of Evidence: Level I, therapeutic study.

## 1. Introduction

Anterior cruciate ligament (ACL) ruptures are among the most common musculoskeletal injuries in athletes, adolescents, and the active population. Arthroscopically assisted ACL reconstruction (ACLR) is one of the top 10 orthopaedic surgical procedures performed annually worldwide [1,2]. Knee braces are often used as external stabilizers for postoperative rehabilitation, although their value remains controversially discussed [3]. Functional braces are designed to provide knee stability while maintaining mobility in the return-to-sports (RTS) period [4,5,6,7,8,9], whereas rehabilitative braces allow early controlled mobilisation immediately after surgery [4,6,8,9,10]. Rehabilitative braces protect the graft from excessive varus and valgus stress, and minimise anterior-posterior translation and rotation between the femur and tibia [11,12,13,14]. Braces may provide subjective stability in the late postoperative phase, especially against mechanical stress [5,15,16]. Various authors also reported a positive effect of rehabilitative braces on proprioception [17,18]. However, improper use of a brace can also lead to secondary damage [19] and muscle atrophy [7], resulting in significant additional costs (Appendix A.1, Economics) [20].

There is conflicting evidence on the effectiveness of postoperative bracing after isolated ACLR [4,9,21,22,23,24,25,26]. Several studies have shown that bracing after isolated ACLR using bone-patellar tendon bone (BTB) autograft does not provide any benefit [7,27,28,29,30]. In contrast, the effect of a totally brace-free rehabilitation after isolated ACRL with hamstring tendon (HT) autograft failed to be shown in previous clinical studies [15,23]. The present studies differ in their design from the current trial, as wearing of an orthotic alternative was omitted in our control group. To date, there is no prospective randomised trial that completely omits the use of any external stabilizer in the early postoperative phase. Confirmative data are needed to substantiate clinical decisions in this scenario [31,32].

Therefore, a randomised trial comparing brace-free and brace-based rehabilitation approaches was designed. The primary outcome measure was the difference in subjective IKDC scores (sIKDC) 12 months after isolated ACLR using HT autograft. The secondary objective was to investigate the isokinetic strength across different muscle groups as a possible measure for monitoring the effectiveness of rehabilitation. It was hypothesised that brace-free rehabilitation is not inferior to brace-based rehabilitation in terms of functional and health-related quality of life outcomes.

## 2. Materials and Methods

### 2.1. Study Design and Oversight

This single-centre randomised controlled non-inferiority trial with parallel-group assignment in a 1:1 ratio was carried out at a tertiary care referral trauma centre in Germany. It was conducted in accordance with the Consolidated Standards of Reporting Trials (CONSORT) and principles of the International Conference on Harmonization Good Clinical Practice (ICH-GCP). The Institutional Review Board (IRB) of the University Witten/Herdecke, Germany, approved the protocol (Reference-No. 14/2015) on 7 July 2015. The trial commenced on 1 June 2016 and was registered at the German Clinical Trial Registry (DRKS00011774).

### 2.2. Patients and Recruitment

Prior to enrolment, patients were informed about the trial purpose and procedures, and provided written informed consent. Patients aged 18–60 years with a body mass index (BMI) < 35 kg/m^2^ scheduled for isolated ACLR of a primary ACL tear using the ipsilateral HT were enrolled. Patients demanding partial meniscal resections and those with smaller meniscal lesions in the white–white zone not requiring resection remained eligible to participate, as were subjects with previous injury to the contralateral knee but normal knee function. Subjects with ligament injuries other than the ACL, previous knee ligament surgeries, severe knee osteoarthritis of Kellgren–Lawrence grade ≥ 3, cartilage replacement procedures, and necessary meniscal repair were excluded.

### 2.3. Interventions

All surgical procedures were performed by two advanced orthopaedic surgeons, using ipsilateral HT autograft. The patients were in a supine position, and an electric leg holder was used. Following diagnostic arthroscopy, the semitendinosus tendon was harvested via an oblique tibial skin incision, and a quadrupled single-bundle graft was then created. Only in cases of a small graft diameter (<7.5 mm), the gracilis tendon was harvested additionally to obtain a six-fold graft. First the femoral tunnel was drilled through the anteromedial portal, respecting the femoral attachment of the torn ACL. The tibial tunnel was drilled in an outside-in technique using a target device and orientating on the tibial origin of the torn ACL. Via a transtibial shuttle device the graft was pulled into the joint. An extracortical button device was used for femoral fixation (Endobutton CL; Smith & Nephew, London, UK). Tibial fixation was performed using a hybrid fixation technique with a bioabsorbable screw (Megafix, Karl Storz) and washer (Endotack, Karl Storz-Endoskope, Tuttlingen, Germany). After wound closure the position of the fixation devices and the drill holes was controlled by X-ray.

An independent research assistant prepared opaque envelopes based on a computer-generated randomisation list. After surgery, the surgeon opened the envelope in operating theatre and assigned patients to the brace-based or brace-free group according to random allocation. Blinding was not possible for either the surgeon or patient because of the possibility of wearing a brace.

**Figure A1:**
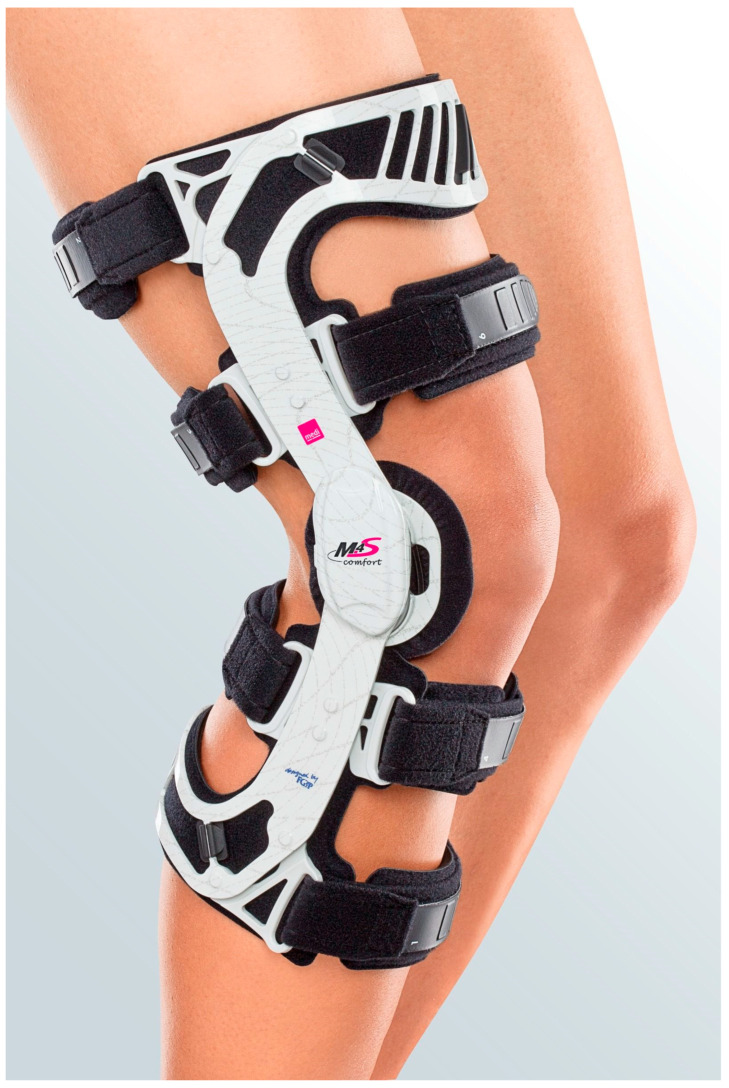
Medi M.4 brace, medi GmbH, Bayreuth, Germany (https://www.medi.de/produkte/m-4s-comfort/, accessed on 4 January 2023).

**Figure A2:**
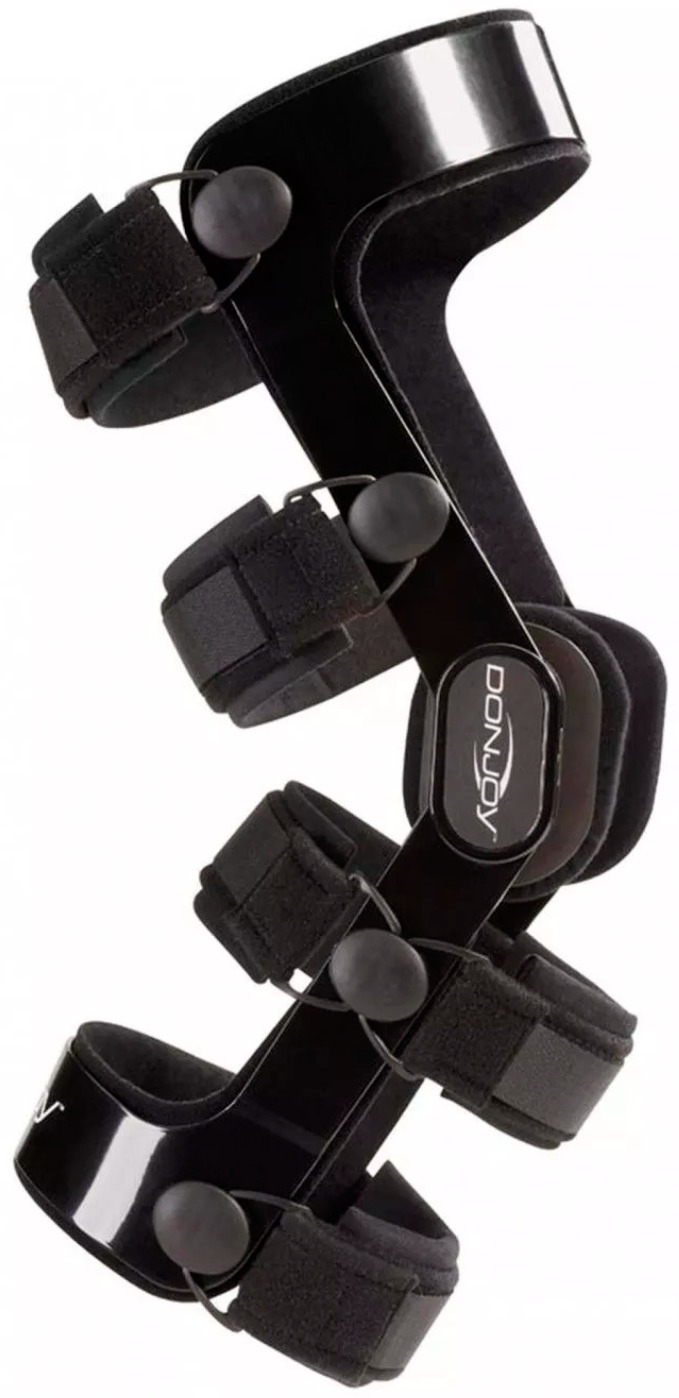
DONJOY 4TITUDE brace, ORMED GmbH, Freiburg, Germany (https://www.ortho-team.ch/produkte/knie-2/knieorthese-donjoy-4titude, accessed on 4 January 2023).

Those in the brace-based group wore a technically identical rehabilitative four-point hard-frame knee brace (Medi M.4, medi GmbH, Bayreuth, Germany (Figure A1), or DONJOY 4TITUDE, ORMED GmbH, Freiburg, Germany (Figure A2)) for six weeks postoperatively. In some cases, patients had already received one of the two mentioned knee braces preoperatively by the family doctor, so that we did not prescribe a new one in order to avoid additional costs. The braces mentioned are two common models that can be used equivalently. Patients in the brace-free group underwent rehabilitation without any external stabilizer.

### 2.4. Perioperative Management and Rehabilitation

All patients underwent the same in-house rehabilitation protocol (Appendix A.2, In-house rehabilitation protocol). It was handed out to all patients postoperatively and explained in detail by one of the in-house physiotherapists. Adherence to the protocol was verified with patients at scheduled follow-up time points. To prevent loss of extension, the operated knee was immobilised in extension splint at night for a maximum of 1 week after surgery in both groups. The standardised rehabilitation protocol aimed at full weight-bearing four weeks after surgery and included consistent near-home physiotherapy for at least four months.

### 2.5. Outcomes

Primary endpoint was the difference in sIKDC [33] 12 months postoperatively. The sIKDC is an effective questionnaire for monitoring patients after ACLR [34] and contains 10 questions regarding symptoms, activities of daily living, sports, and knee function.

Secondary subjective outcomes included Lysholm and Tegner activity scales. The Lysholm score records knee-specific physical function, pain, and symptoms in eight domains on a scale from 0–100, with 100 indicating unlimited function [35]. The Tegner activity scale rates the degree of knee-specific activity on a scale from 0–10, with 10 representing the highest possible activity level [36].

Health-related quality of life was assessed using the physical (PCS) and mental component summary (MCS) scores of the Short Form 36 (SF-36) questionnaire [37]. The SF-36 contains 36 items with questions on physical functioning and mental health, with a scale value between 0 and 100 (the best value being 100).

Subjective confidence in RTS activities was evaluated using the Anterior Cruciate Ligament—Return to Sport after Injury (ACL-RSI) score. The ACL-RSI score, with values ranging from 1 to 10, includes 5 questions on emotional well-being, 5 questions on confidence in physical performance, and 2 questions on risk assessment. Higher scores indicate a more positive psychological response. The total score was determined by adding the values of the 12 responses and then calculating their relationship to 100 to obtain a percentage.

Secondary objective outcomes included the objective IKDC score (oIKDC) [38]. The oIKDC obtains seven measurement points (knee examination, graft harvest morbidity, and radiographic findings) categorized into groups A through D, with A representing the best group. Further secondary outcome parameters were thigh muscle strength tested by isokinetic dynamometry as well as instrumentally measured anterior tibial translation (knee laxity tester (KLT), Karl Storz-Endoskope, Tuttlingen, Germany; (mm)) at 30° knee flexion in the supine (Lachman) position.

**Figure 1:**
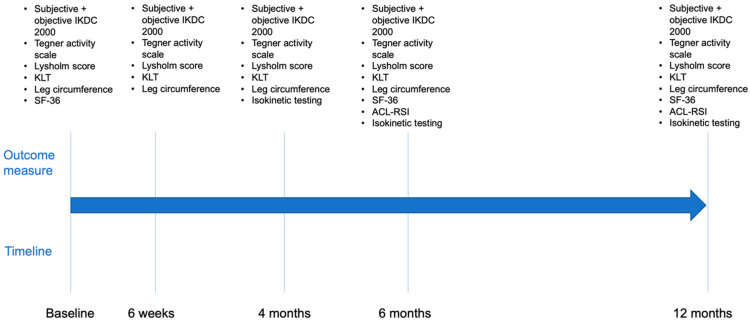
Timeline of follow-up examinations. ACL-RSI, Anterior Cruciate Ligament—Return to Sport after Injury. IKDC, International Knee Documentation Committee. KLT, knee laxity tester. SF-36, Short Form 36.

Baseline data were measured at outpatient clinic before, 6 weeks, 4, 6, and 12 months after the index procedure (Figure 1). All measurements were carried out independently by two experienced investigators. Isokinetic strength testing was conducted by two experienced sports scientists at 4, 6, and 12 months.

### 2.6. Isokinetic Strength Testing

Isokinetic strength testing was conducted on a Biodex System 3 device (Biodex Medical Systems, Inc., Shirley, New York, NY, USA). Isokinetic testing proved good to high test–retest reliability and has extensively been employed as a safe method to determine muscle strength in patients after ACLR [39,40,41,42]. Isokinetic torque data, corrected for gravity, were collected at a frequency of 100 Hz and an angular velocity of 60°/s (four repetitions) [43,44]. Maximal effort torque data from four repetitions for each participant were analysed by two independent researchers. Data were normalised to body weight, reported as mean relative peak torque, and filtered by a second order 5 Hz Butterworth low-pass bidirectional filter [45].

### 2.7. Statistical Analysis

Data were described according to their quality and expression as numbers, frequencies (%), means, or medians, with suitable measures of distribution (e.g., ranges, interquartile ranges (IQRs), and standard deviations (SDs)). Whenever possible, 95% confidence intervals (CIs) were calculated and reported for individual estimates, differences, and ratios. Primary endpoint was the sIKDC 12 months postoperatively. We assumed non-inferiority of brace-free rehabilitation compared to brace-based rehabilitation after isolated ACLR operationalized by the difference in sIKDC scores after 12 months of follow-up. According to the American Orthopaedic Society for Sports Medicine (AOSSM) Outcomes Task Force, the reported responsiveness of the sIKDC ranges from 6.7 to 20.5 in the Minimal Detectable Change (MCD) and 3.2 to 16.7 in the Minimal Clinically Important Difference (MCID) [46]. We simulated several scenarios with varying MCD and MCID using conservative type I (two-sided 0.05) and type II (0.2) errors. This leads to an estimated power of 80%. The calculations were made with Power Analysis & Sample Size (PASS 2020 Version 20.0.6 (NCSS LLC, East Kaysville, UT, USA)). The most efficient, clinically reasonable, and methodologically sound trial set-up was reached with a non-inferiority margin of 9.0 points, demanding complete data from 68 patients per trial arm. Given a presumed drop-out-rate of 10% and loss-to-follow-up-rate of 15%, we attempted to randomize 106 patients. A generalised linear mixed model (GLMM) was used for primary endpoint analysis. We reported central estimates (i.e., beta coefficients) with 95% CI which must include the null to prove the non-inferiority hypothesis. Secondary outcomes were explored graphically, and either by repeated-measures ANOVA or GLMM. STATA 16.1 (StataCorp, College Station, TX, USA) was employed for all analyses (Appendix A.3, Statistical analysis).

## 3. Results

**Figure 2:**
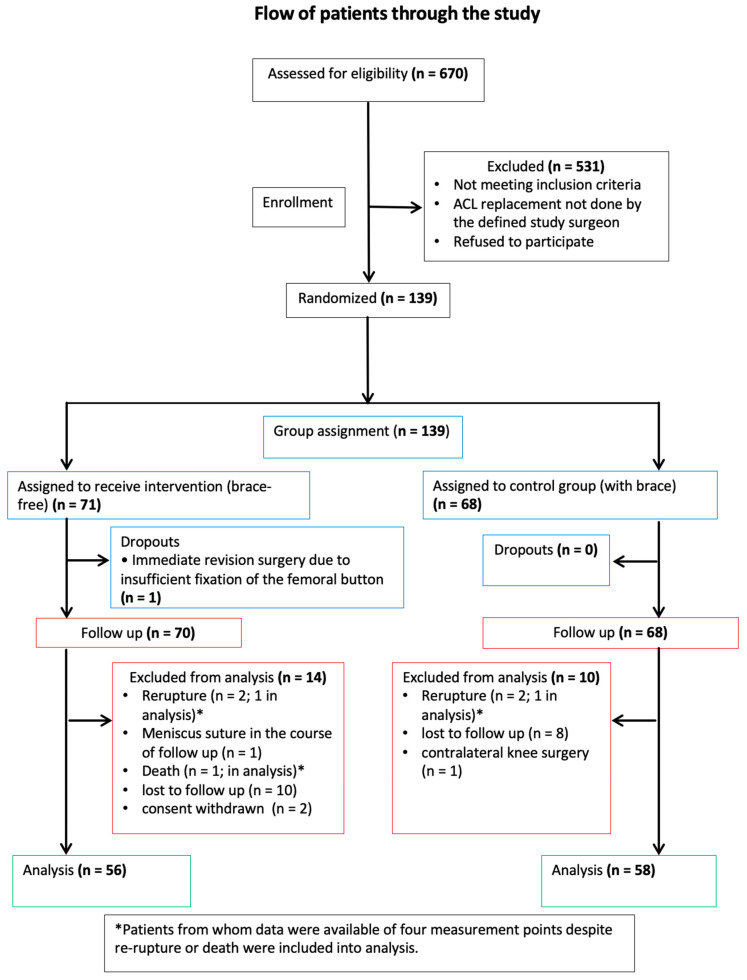
Patient inclusion flowchart. ACL, anterior cruciate ligament.

Of 670 patients screened, 531 did not meet inclusion criteria. A total of 139 patients were recruited and randomised (71 in the intervention group and 68 in the control group) from June 2016 to April 2019 (Figure 2). One patient in the intervention group dropped-out post-randomisation because of immediate revision surgery due to insufficient fixation of the femoral button. A total of 70 (intervention group) and 68 (control group) patients were included in the analysis. Fourteen patients in the intervention group and 10 patients in the control group were excluded from analysis for the following reasons: (1) lost to follow-up (10 versus 8), (2) withdrawal of consent (2 versus 0), adverse event (2 versus 1), and contralateral knee operation during follow-up (0 versus 1).

### 3.1. Baseline Data

Baseline demographic and clinical characteristics of patients are shown in Table 1. Both groups were similar in all categories, and no clinically significant differences were observed (n.s.). A total of 20 patients showed a stable meniscus lesion during arthroscopic examination, which did not require suturing (brace-based group: medial 4 superficial lesions, 3 small flap lesions, 1 small lesion in the white–white zone not requiring treatment; lateral 3 superficial lesions, 1 small flap lesion; brace-free group: medial 2 small flap lesions, 1 superficial lesion; lateral 3 superficial lesions, 2 small flap lesions). In 19 of these lesions, only partial resection was performed, which had no influence on the postoperative rehabilitation plan. The sports level of both groups is shown in Table 2. Listed is the level, activity, and weeks until RTS. Pre-existing injuries and previous interventions on the contralateral side are illustrated in Table 3. In 9 cases, ACLR had been performed on the contralateral side.

Complications occurring post-randomisation are listed in Table 4.

### 3.2. Primary Outcome Measure

**Figure 3:**
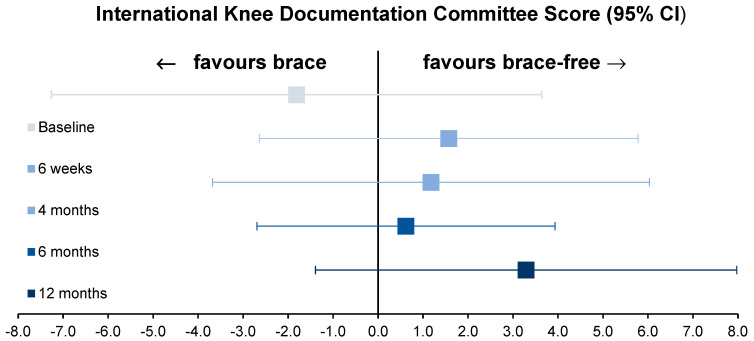
Mean difference of the subjective International Knee Documentation Committee Score values (95% CIs) during the observation interval.

Brace-free rehabilitation proved to be non-inferior to brace-based rehabilitation based on an a priori-defined MCID of 9.0 (mixed-effects regression coefficient 1.57, 95% CI −2.56–5.70, *p* for non-inferiority < 0.0001). Figure 3 shows the mean differences in the sIKDC values with 95% CIs during the observation interval. With the above-mentioned lowest MDC and MCID, *p* values for non-inferiority remained at 0.0254 and 0.0001, respectively. Adhering to a threshold of 0.05, apart from discussions of lowering the level of significance to 0.005 (to avoid false-positive trial findings) and assuming minimal non-inferiority margins, it is statistically unlikely that our observations are compatible with chance.

### 3.3. Secondary Outcome Measures

**Figure 4:**
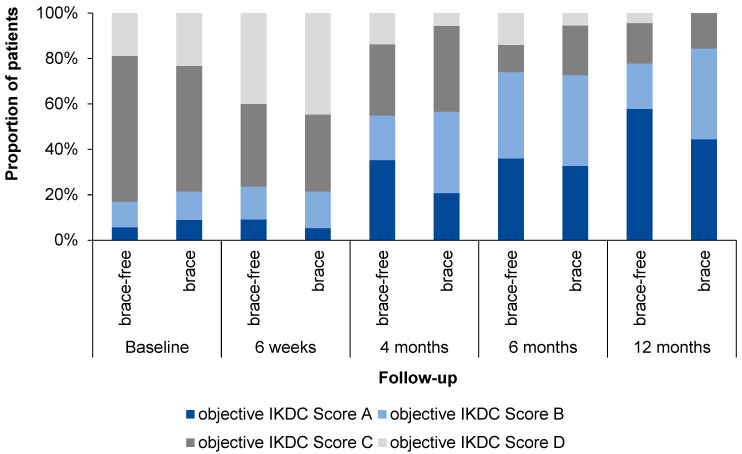
The objective International Knee Documentation Committee grading.

The results of the oIKDC are shown in Figure 4. Remarkably, the proportion of patients with normal oIKDC grades A increased earlier and faster during rehabilitation within the brace-free group.

The ACL-RSI improved from 70.2 ± 18.1 points in the brace-free and 65.9 ± 19.8 points in the brace-based group after 6 months up to 70.4 ± 23.3 points in the brace-free group and 72.5 ± 20.4 points in the brace-based group after 12 months. A cut-off value of 56 points is reported [47] to reflect readiness to RTS, thus both groups rated their ACL injury related self-confidence high enough to participate in their pre-injury sporting activities. Relative knee instability was calculated as the difference between the KLT measured anterior tibial translation of the unaffected and affected knee. Brace-free rehabilitation proved to be non-inferior to brace-based rehabilitation (mixed-effects regression coefficient 0.64, 95% CI −0.01 to 1.28, *p* for non-inferiority < 0.0001).

The mean Lysholm score at 12 months was 88 ± 4.0 and 85 ± 3.9 for the brace-based and brace-free groups, respectively. Hence, brace-free rehabilitation was non-inferior to brace-based rehabilitation, given a priori-defined MCID of 9.0 (with a mixed-effects regression coefficient of 0.37, 95% CI −6.12 to 5.38, *p* < 0.0001). With the lowest MDC and MCID of 8.9 and 10.1, as provided by the AOSSM Outcomes Task Force, *p* values for non-inferiority remained at 0.0044 and 0.0012, respectively.

The mean Tegner activity scale value increased from 2.7 ± 0.6 (brace-free group) and 3.5 ± 0.6 (brace-based group) prior to injury to 5.3 ± 0.6 and 5.5 ± 0.5 at 12 months after surgery, respectively. Brace-free rehabilitation proved to be non-inferior to brace-based rehabilitation (mixed-effects regression coefficient −0.055, 95% CI −0.59 to 0.70, *p* for non-inferiority, <0.0001). Furthermore, brace-free rehabilitation was also found to be non-inferior to brace-based rehabilitation when judged based on the health-related quality of life measurements (mixed-effects regression coefficient −0.258, 95% CI −4.40 to 3.89, *p* for non-inferiority < 0.0001) and mean MCS scores (mixed-effects regression coefficient 3.80, 95% CI −1.57 to 9.17, *p* for non-inferiority < 0.0001).

Data on Lysholm and Tegner scores, KLT measurements, and SF36 subscale scores for all follow-up timepoints are shown in Table 5.

### 3.4. Isokinetic Strength Testing

**Figure 5:**
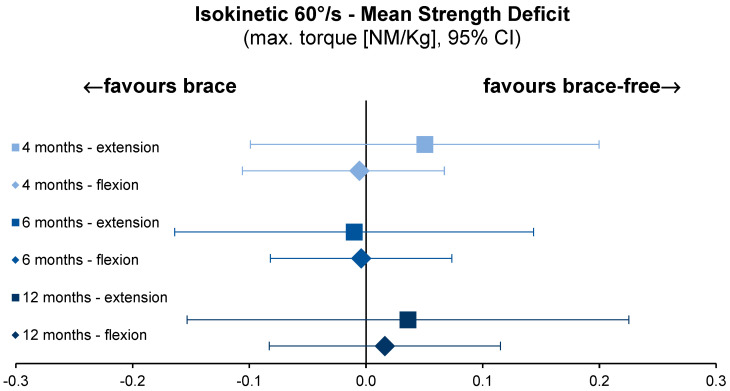
Mean strength deficit between ACLR and uninvolved leg, normalised to individual bodyweight for knee extension and flexion torque at 60°/s isokinetic testing velocity. ACLR, anterior cruciate ligament reconstruction. kg, kilogram. max., maximal. NM, newton meter. s, second.

Force production of the knee extensors and knee flexors was measured at an angular velocity of 60°/s. Maximal torque for the knee extensors increased equally in both groups from 4 to 12 months after surgery. The same trend was observed for the maximal knee flexion torque at these time-points (Figure 5). The mixed-effects regression coefficient (0.0199, 95% CI −0.196–0.2361, *p* for non-inferiority < 0.0001) revealed no significant difference between the groups for knee extension and knee flexion (−0.0458, 95% CI −0.1505–0.059, *p* < 0.0001).

To differentiate strength differences between the brace-free and brace-based rehabilitation groups, the mean group differences for all isokinetic parameters were calculated with reference to the ACLR knee.

## 4. Discussion

The most important finding of this study was, that subjective and objective outcomes following isolated ACLR did not differ significantly between subjects wearing a brace and those not wearing a brace postoperatively. Therefore, the hypothesis of non-inferiority of brace-free versus brace-based rehabilitation was proven. Moreover, instrumented laxity measurements and isokinetic knee strength in the brace-free group proved to be non-inferior to the brace-based group. Finally, both groups rated their ACL injury related self-confidence high enough to participate in pre-injury sporting activities.

Although many consider bracing an integral part of rehabilitation after ACL injuries [36,48,49], Level I data after isolated ACLR using HT autograft is still unclear. Several studies have investigated the effect of functional and rehabilitative bracing after isolated ACLR using BTB autograft [7,27,29,30,50,51,52,53]. Two systematic reviews suggest that bracing must not be recommended after BTB autograft ACLR. The authors highlighted several methodological limitations such as inappropriate sample size calculation, unclear randomisation schemes, and selection bias [54,55].

Rehabilitation after HT-ACLR differs from that after BTB autografts as there is no interface between bone and tendon, resulting in a prolonged remodelling process following ACLR [56]. Therefore, less aggressive rehabilitation protocols are advised [57]. Hence, it is not surprising that ACL surgeons still recommend bracing after isolated ACLR using HT autograft, according to recent surveys [31,32]. Only two randomised clinical trials have investigated the effect of bracing after isolated ACLR using HT autograft [15,23]. In contrast to the current study, Birmingham et al. [15] compared functional bracing versus neoprene sleeves only starting 6 weeks after surgical intervention, rather than examining rehabilitative bracing directly in the first 6 weeks after surgery. Functional bracing did not improve primary and secondary outcomes after 12 and 24 months as compared to neoprene sleeves, however a different time point of bracing was considered compared to the current study. Mayr et al. [23] performed a study comparing a rehabilitative brace versus a water-filled soft brace for 6 weeks postoperatively. The soft brace group showed significantly higher Tegner activity, Lysholm, and sIKDC scores 6 and 12 months postoperatively. Both studies did not completely omit the wearing of an orthotic alternative in the control group (neoprene sleeve, water-filled soft brace). In contrast, in the current study no external stabilizer was used in the control group in order to test against a truly brace-free rehabilitation protocol. Furthermore, the previous studies only considered clinical measurements and questionnaires with no comparison of muscle strength using isokinetic strength testing. This secondary outcome parameter is an important strength of the current study, as it could be shown that there is no difference in terms of quantitative muscle strength in both groups.

In addition, the present trial provides strong evidence that bracing after isolated ACLR using HT autograft does not lead to superior functional outcomes compared to brace-free rehabilitation.

Patients in either group reported comparable results (70.4 ± 23.3 point in the brace-free group and 72.5 ± 20.4 in the brace-based group) in the ACL-RSI, rating readiness to RTS after injury [58].

As recently highlighted, studies that minimise bias are needed to further evaluate the role of bracing in ACL injuries [3,54]. In the current trial, only patients with isolated ACLR not requiring reconstruction of meniscal tears, treatment of chondral lesions, or treatment of additional ligament tears were included. To minimise performance and detection bias, surgeons and patients were kept blinded to treatment assignment until the end of surgery. Additionally, all ACLR were performed by two experienced surgeons, all examinations were performed by only two experienced investigators, and isokinetic strength testing was performed by two experienced sport scientists. Moreover, a low rate of attrition was achieved, with only 18/139 participants lost to follow-up.

This study is not without limitations. A significant number of patients required combined ACLR and meniscal suturing. As we excluded these patients, it remains unclear whether our results are applicable to rehabilitation after any type of combined ACLR surgery, thereby hampering external validity. Additionally, we herein report results 12 months after surgery and cannot make predictions whether our findings are sustainable. As however the early postoperative period was assessed, a longer follow-up would have been outside the aim of the current investigation. In our collective, a male predominance was seen. This is in line with other studies on ACLR, as the incidence of ACL injury is higher in men in the general population.

According to the results of this study, the use of any external stabilizers after isolated ACLR using HT autograft should be avoided in clinical practice. The results reduce treatment costs and simplify the rehabilitation protocol, which partly varied up to now.

## 5. Conclusions

Brace-free rehabilitation after isolated ACLR using HT autograft is non-inferior to brace-based protocols regarding physical recovery one year postoperatively. Patients without bracing after isolated ACLR showed no significant difference in the ACL-RSI compared to the control group in the current study. Thus, it can be concluded that patients regained equal confidence in the operated knee after 6 and 12 months, regardless of the use of a brace. It remains questionable whether bracing can also be avoided after revision and combined ACLR.

## Figures and Tables

**Table 1:** Baseline characteristics of patients allocated to the brace-free and brace-based study groups. Values represent means (standard deviations) unless stated otherwise. One patient (with brace) underwent both medial and lateral meniscus resection.

Characteristics	Brace	Brace-Free	*p* Value
Demographics	*n* = 58	*n* = 56	
Male gender, *n* (%)	37 (63.8)	37 (66.1)	0.846 *
Right knee, *n* (%)	28 (48.3)	26 (46.4)	0.854 *
Age (years), mean (SD)	33.2 (12.3)	31.5 (10.8)	0.429 †
Body mass index (kg/m^2^), mean (SD)	26.0 (3.1)	25.9 (4.1)	0.881 †
Smoking, *n* (%)	12 (20.7)	17 (30.4)	0.285 *
Interval between injury and surgery (days), mean (SD)	109.2 (199.5)	123.4 (257.4)	0.744 †
**Cartilage damage**			
Medial femoral, *n* (%)	11 (19.3)	12 (21.43)	0.819 *
Lateral femoral, *n* (%)	2 (3.51)	0 (0.00)	0.496 *
Medial tibial, *n* (%)	5 (8.77)	5 (8.93)	1.000 *
Lateral tibial, *n* (%)	3 (5.26)	2 (3.57)	1.000 *
**Meniscal lesion**			
Meniscus medial			
Lesion, *n* (%)	8 (13.79)	3 (5.36)	
Meniscus lateral			
Lesion, *n* (%)	4 (6.9)	5 (8.93)	
Meniscus resection during ACLR, *n* (%)	11 (19.3)	8 (14.29)	0.799 *
Meniscus medial, *n* (%)	7 (12.07)	3 (5.36)	0.322 *
Meniscus lateral, *n* (%)	4 (6.9)	5 (8.93)	0.74 *

* Fisher’s exact test; † *t* test, unequal variances. ACLR, anterior cruciate ligament reconstruction. kg, kilogram. m, meter. SD, standard deviation.

**Table 2:** Sports level activity preoperative and return to sports after rehabilitation.

Sports Level	*n*	Brace	Brace-Free
Professional	4	*n* = 0	*n* = 4
Activity (%), mean (SD)			75 (35.36)
Return to sports (w), mean (SD)			22 (8.49)
**Amateur**	**47**	**n = 28**	**n = 19**
Activity (%), mean (SD)		73.86 (35.72)	57.65 (40.04)
Return to sports (w), mean (SD)		31.95 (36.08)	28.67 (15.44)
**Recreational sports**	**52**	***n* = 27**	***n* = 25**
Activity (%), mean (SD)		55.23 (45)	61.25 (40.97)
Return to sports (w), mean (SD)		31.62 (16.36)	33.27 (18.03)
**No sport**	**7**	***n* = 3**	***n* = 4**

SD, standard deviation. w, weeks.

**Table 3:** Pre-existing injuries and previous interventions on the contralateral side.

Pre-Existing Injuries and Previous Interventions Contralateral Side	Brace	Brace-Free	Total
none	58	65	123
ACL reconstruction	5	4	9
ACL rupture, conservative	1	0	1
Rupture of the collateral ligaments, conservative	1	1	2
Meniscus/cartilage lesion	1	1	2
Patella luxation	1	0	1
Posttraumatic arthrosis	1	0	1
**Total**	**68**	**71**	**139**

ACL, anterior cruciate ligament.

**Table 4:** Complications post-randomisation.

Complications	Brace	Brace-Free	Total
none	62	59	121
Re-rupture	2	2	4
Cyclops, operative treated	4	4	8
Postoperative joint lavage, non-infectious hemarthrosis	0	1	1
Dislocated screw fragment	0	1	1
New Meniscus lesion, operative suture	0	1	1
Revision button fixation	0	1	1
Plica resection	0	1	1
Notchplastic in case of ACL impingement	0	1	1
**Total**	**68**	**71**	**139**

ACL, anterior cruciate ligament.

**Table 5:** Secondary outcome of Lysholm and Tegner Score, KLT measurements, SF36-MCS and PCS subscales.

Timepoint	Group	Lysholm Score, Mean ± SD	Tegner Score, Mean ± SD	Knee Laxity Measurement [mm], Mean ± SD	SF 36-MCS Scale, Mean ± SD	SF 36-PCS Scale, Mean ± SD
**Baseline**	Brace	54.9 ± 4.7	3.5 ± 0.6	−1.0 ± 0.5	51.6 ± 2.8	38.5 ± 2.3
	Brace-free	56.0 ± 5.1	2.7 ± 0.6	−0.9 ± 0.6	49.5 ± 3.2	39.2 ± 2.5
**6 weeks**	Brace	60.9 ± 4.7	2.5 ± 0.4	0.2 ± 0.5	52.8 ± 4.2	37.8 ± 3.7
	Brace-free	61.2 ± 5.2	2.5 ± 0.4	−0.4 ± 0.4	46.5 ± 6.7	36.2 ± 3.9
**4 months**	Brace	78.3 ± 4.3	3.8 ± 0.4	−0.8 ± 0.5	58.3 ± 1.8	45.3 ± 4.6
	Brace-free	75.1 ± 5.2	3.6 ± 0.5	−0.4 ± 0.4	50.9 ± 5.0	45.9 ± 3.9
**6 months**	Brace	84.3 ± 2.4	4.6 ± 0.5	−0.3 ± 0.5	54.8 ± 1.7	50.4 ± 1.3
	Brace-free	83.5 ± 2.7	4.2 ± 0.5	−0.8 ± 0.5	52.6 ± 2.4	51.0 ± 1.4
**12 months**	Brace	88.3 ± 4.0	5.5 ± 0.5	−0.4 ± 0.5	52.9 ± 1.4	52.9 ± 1.4
	Brace-free	85.1 ± 3.9	5.3 ± 0.6	−0.6 ± 0.4	52.3 ± 2.0	52.3 ± 2.0

SD, standard deviation. SF-36, Short Form 36: MCS, mental component summary. PCS, physical component summary.

## Data Availability

The data presented in this study are available on request from the corresponding author.

## Appendix A

### Appendix A.1. Economics

The economic burden of current prescription practice can be quantified by calculating annual cost for orthotic prescription. Data on the German population estimates ACL injury incidence rates of 46 per 100.000 person years. In Germany the actual cost of orthotic prescription can be estimated at 450 EUR to 600 EUR for a rigid hard frame brace, depending on model specifications [59]. Assuming a population of 83.1 M people for Germany, providing braces for approximately 38.180 ACL injuries would amount to annual costs of up to 17.1 million to 22.9 million EUR. This approximation does not account for complex ACL injuries where orthotic prescription might be mandatory. However, following the above stated numbers, a brace-free rehabilitation after isolated ACLR could reduce cost in public healthcare of several million Euros annually.

### Appendix A.2. In-House Rehabilitation Protocol

	**Operating Day**	**Day 1**	**Day 3**	**Day 14**	**Day 28**	**Week 7**	**Month 4**	**Month 7**	**Month 9**
Weight bearing	20 kg partial load			Load increase pain-adapted	Full weight bearing				
Splint/Brace		Rehabilitative four-point hard-frame knee brace				Training off the brace			
Physiotherapy	Free mobility in the ankle joint	Motor rail 0-0-90° Muscle stimulation Isometry quadriceps Mobilisation with 2 crutches	Motor rail 0-0-90° Patellar mobilisation Active knee flexion/Passive knee extension		Coordination training Proprioception training	Muscle training			
Sports/Other		Redon removal if necessary Bandage change		Suture removal		Aqua jogging Crawl swimming Cross trainer Ergometer Nordic walking	Jogging Jump training	Contact sport	Competitive sports High risk sport (Ski Alpine, Squash)

kg, kilogram.

### Appendix A.3. Statistical Analysis

The general equation for claiming non-inferiority reads

(A1) $$T_{L}=\frac{{\hat{\mu }}_{E}-{\hat{\mu }}_{S}+\delta }{\sqrt{S_{p}^{2}\left(\frac{1}{n_{E}}+\frac{1}{n_{S}}\right)}}$$

where

$T_{L}$ is the test statistic

${\hat{\mu }}_{E}$ is the mean IKDC in the experimental (brace) group

${\hat{\mu }}_{S}$ is the mean IKDC in the standard (brace-free) group

δ is the mean difference in IKDC between the brace and control group defining non-inferiority

$S_{p}^{2}$ is the pooled standard deviation

$n_{E}$ is the sample size in the experimental (brace) group

$n_{S}$ is the sample size in the standard (brace-free) group

For claiming non-inferiority based on regression estimates from a generalised linear mixed model (GLMM), the equation reads

(A2) $$T_{L}=\frac{{\hat{\beta }}_{1}+\delta }{{\hat{SE}}_{\beta_{1}}}$$

where

$T_{L}$ is the test statistic

${\hat{\beta }}_{1}$ is the slope of the regression line for the experimental compared to the control group (reference) without covariates

δ is the mean difference in IKDC between the brace and control group defining non-inferiority

${\hat{SE}}_{\beta_{1}}$ is the standard error of the slope

We reported central estimates (i.e., *beta* coefficients) with 95% CI which must include the null to prove the non-inferiority hypothesis. Secondary endpoints were explored graphically, repeated measures ANOVA, and GLMM regression.
